# The DDR1 Tyrosine Kinase Promotes Th17 Cell Migration in Three-Dimensional Collagen and into the Joints During Inflammatory Arthritis

**DOI:** 10.3390/ijms27136043

**Published:** 2026-07-06

**Authors:** Chakib Hamoudi, Mehdi Toghi, Anahita Lashgari, Fawzi Aoudjit

**Affiliations:** 1 Division of Infectious and Immune Diseases, CHU de Quebec-Université Laval Research Center, CHUL, Quebec, QC G1V 4G2, Canada; 2 ARThrite Research Center, Laval University, Quebec, QC G1V4G2, Canada; 3 Department of Microbiology-Infectiology and Immunology, Faculty of Medicine, Laval University, Quebec, QC G1V 0A6, Canada

**Keywords:** Th17, cell surface molecules, cell trafficking, arthritis

## Abstract

Th17 cells play an important role in adaptive immunity and inflammation; however, the mechanisms regulating their migration into inflammatory tissues are not fully understood. In this study, we found that ex vivo human effector/memory but not central/memory Th17 cells express and use the discoïdin domain receptor 1 (DDR1) to migrate in collagen gels. The tyrosine kinase activity of DDR1 is essential to this process since its blockade with the DDR1 kinase inhibitor 7rh and the DDR1 kinase-dead construct inhibited the migration of human Th17 cells. Our results indicated that the DDR1 kinase activity enhanced Th17 cell migration by activating the MAPK/ERK pathway. We then examined the role of DDR1 in the inflammatory model of collagen-induced arthritis (CIA). Treatment of CIA mice with the DDR1 kinase inhibitor 7rh led to reduced arthritis severity, synovial inflammation and cartilage destruction. DDR1 expression is higher on T cells isolated from CIA mice than on those from naïve mice, and its blockade inhibited Th17 cell infiltration into the joints, which was associated with the inhibition of IL-17 levels. Along these lines, isolated T cells from 7rh-treated arthritic mice showed a drastic reduction in migration in collagen gels compared to those isolated from control arthritic mice. Further, the 7rh treatment reduced the levels of TNF-α and IL-1β and increased the levels of IL-10, thus promoting an anti-inflammatory environment. Our findings provide an important role for the DDR1 tyrosine kinase in promoting Th17 cell infiltration into inflammatory tissues, especially in collagen-rich tissues like the joints, and in the development of arthritis.

## 1. Introduction

T cell migration into peripheral tissues is a critical step in adaptive immune response and inflammatory diseases. To reach their target sites and exert their functions, effector T cells migrate through the vascular endothelium and the extracellular matrix (ECM) of the interstitial tissue in which collagen is a major matrix protein. Hence, interactions of effector T cells with collagen are likely to regulate their trafficking and localization and therefore could play a key role in T cell-mediated immunity and inflammation. Leukocytes, including effector T cells, use the amoeboid movement to migrate in three-dimensional (3D) collagen models such as collagen gels, which mimic the matrix organization within the tissues. This movement occurs independently from strong adhesion, integrins and matrix remodeling by metalloproteinases and relies on contact guidance and squeezing through existing gaps [1,2,3,4,5,6]. These studies suggest that additional collagen receptors might be important in T cell migration in collagen.

Discoidin domain receptors (DDR1 and DDR2) are tyrosine kinase transmembrane receptors that are activated by different types of collagen and play important roles in cell proliferation, adhesion, and migration [7,8]. They are involved indirectly in cell adhesion to collagens through the activation of β1 integrin adhesion receptor [9,10,11]. DDR1 is implicated in various pathological conditions including fibrosis [12,13], kidney inflammation [14,15], cardiometabolic disease [16] and cancer invasion and progression [17,18,19].

DDR1 is induced during T cell activation [20,21,22] and its overexpression enhances monocyte migration in 3D collagen [20]. We reported that DDR1 promoted the migration of human polarized Th17 cells in 3D collagen as well as the migration of Th17 cells in the mouse air pouch model [23]. However, it remains unclear if the DDR1 tyrosine kinase activity is required for Th17 cell migration. Indeed, DDR1 can signal independently from its kinase activity as it promotes the formation of invadosomes in tumor cells [24] and the reactivation of breast cancer multi-organ site metastasis [25] without implicating its kinase activity. Similarly, tumor DDR1 does not require kinase activity to promote collagen fiber alignment to exclude immune cells from penetrating the tumors [26].

Despite the previous studies, the role of DDR1 in T cell migration during T cell-mediated immunity remains unknown. We investigated this issue in an animal model of rheumatoid arthritis (RA). RA is an inflammatory disease affecting the joints in which Th17 cells migrate and orchestrate the inflammatory response, leading to cartilage and bone damage [27,28,29]. Given the role of Th17 cells and the abundance of collagen in the arthritic joints [30,31], we hypothesized that by enhancing Th17 cell migration in collagen, DDR1 can play a crucial role in the development of inflammatory arthritis.

In this study, we found that ex vivo human Th17 cells comprising Th17, Th17/Th1 and Th17.1 (non-classical Th1) subsets, which are involved in immunity and autoimmune arthritis [28,32,33,34], express DDR1 and the blockade of the DDR1 tyrosine kinase activity inhibited their migration in 3D collagen. Furthermore, inhibition of DDR1 with the DDR1 tyrosine kinase inhibitor 7rh reduced the development of collagen-induced arthritis (CIA) in mice by affecting Th17 cell infiltration into the joints. Collectively, these results indicate that the DDR1 tyrosine kinase activity is a key pathway of effector T cell infiltration into inflammatory tissues, especially those enriched in collagen, such as arthritic joints.

## 2. Results

### 2.1. DDR1 Is Expressed on Human Effector Th17 Cells

In this study, we examined DDR1 in CCR6^+^CD161^+^CD25^−^ Th17 cells from the peripheral blood of healthy donors that we isolated through cell sorting and flow cytometry. As expected, these cells, which play critical roles in immunity and autoimmune arthritis, contain classical Th17 cells as well as ex-Th17 cells (Th17/Th1 and Th17.1) as determined by the production of IL-17 and IFN-γ (Figure S1).

**Figure 1:**
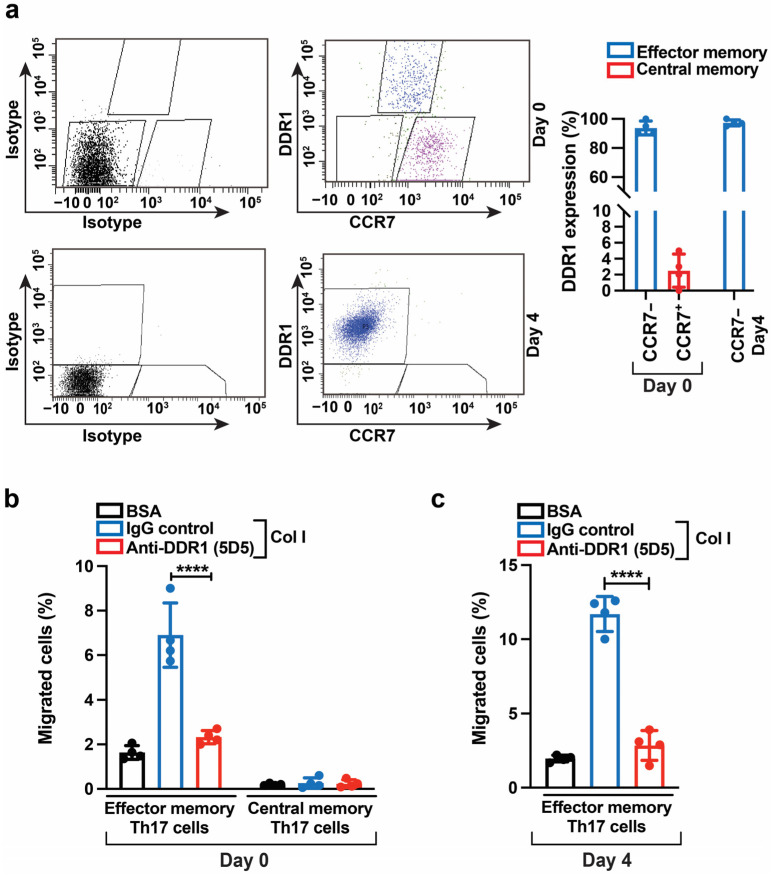
DDR1 is associated with the migration of human effector/memory CD161^+^CCR6^+^CD25^−^ Th17 cells in 3D collagen. The cells were isolated as described in the Materials and Methods section. (**a**) Human effector/memory Th17 cells (CD161^+^CCR6^+^CD25^−^CCR7^−^) but not central/memory Th17 cells (CD161^+^CCR6^+^CD25^−^CCR7^+^) express DDR1. Human CD161^+^CCR6^+^CD25^−^ Th17 cells were FACS-sorted from the peripheral blood of healthy donors and stained either immediately (day 0) or after four days of in vitro expansion (day 4) with isotype control antibodies or with antibodies against DDR1 and CCR7. The FACS plot shown is representative of four independent experiments performed with T cells from four different blood donors. The histogram on the right represents mean ± SD of four different blood donors. (**b**) Effector/memory cells but not central/memory Th17 cells actively migrate through collagen gels (Col I). Cells were treated with either 10 μg/mL of blocking anti-DDR1 antibody (clone 5D5) or control IgG for 1 h at 4 °C. The cells were washed and seeded onto collagen gel-coated inserts (Col). Non-treated cells migrating through BSA-coated inserts were included as a control (BSA). After 24 h, migrated cells were collected from the lower chamber and counted as described in the Materials and Methods section. (**c**) The blocking anti-DDR1 antibody (5D5) inhibits the migration of in vitro expanded human CD161^+^CCR6^+^CD25^−^ Th17 cells. After four days of expansion, the cells were pre-treated with either anti-DDR1 (5D5) or control IgG for 1 h. They were washed and subsequently tested for their migration in collagen gels (Col I). Non-treated cells migrating through BSA-coated inserts were included as a control (BSA). Data represent mean values ± SD of four different experiments performed in triplicate using Th17 cells isolated from four different blood donors. **** *p* < 0.001; calculated using two-way (**b**) or one-way (panel (**c**)) ANOVA with Bonferroni correction.

We first assessed the expression of DDR1 and used CCR7 as a marker to distinguish between central and effector/memory Th17 cells. Flow cytometry analysis of freshly isolated CCR6^+^CD161^+^CD25^−^ Th17 cells (day 0) revealed that DDR1 is preferentially expressed on CCR7-negative Th17 cells corresponding to the effector/memory Th17 cells (Figure 1a). We then sorted CCR7^+^ and CCR7^−^ Th17 cells (Figure S2) and tested their migration potential. As expected, effector/memory Th17 cells are migratory in collagen gels and the blocking anti-DDR1 antibody 5D5 [35] strongly reduced their migration (Figure 1b). In contrast, central/memory Th17 cells, which do not express DDR1, are poorly migratory (Figure 1b). In support, freshly isolated CCR6^+^CD161^+^CD25^−^ Th17 cells expanded with CD3/CD28 beads for four days transitioned to an effector/memory phenotype, with almost all the cells becoming DDR1-positive (Figure 1a). These cells also migrate in collagen gels and the blocking anti-DDR1 antibody reduced their migration (Figure 1c). These results indicate that DDR1 is associated with human effector/memory Th17 cells and their migration in 3D collagen.

### 2.2. DDR1 Kinase Is Required for Human Th17 Cell Migration in 3D Collagen

**Figure 2:**
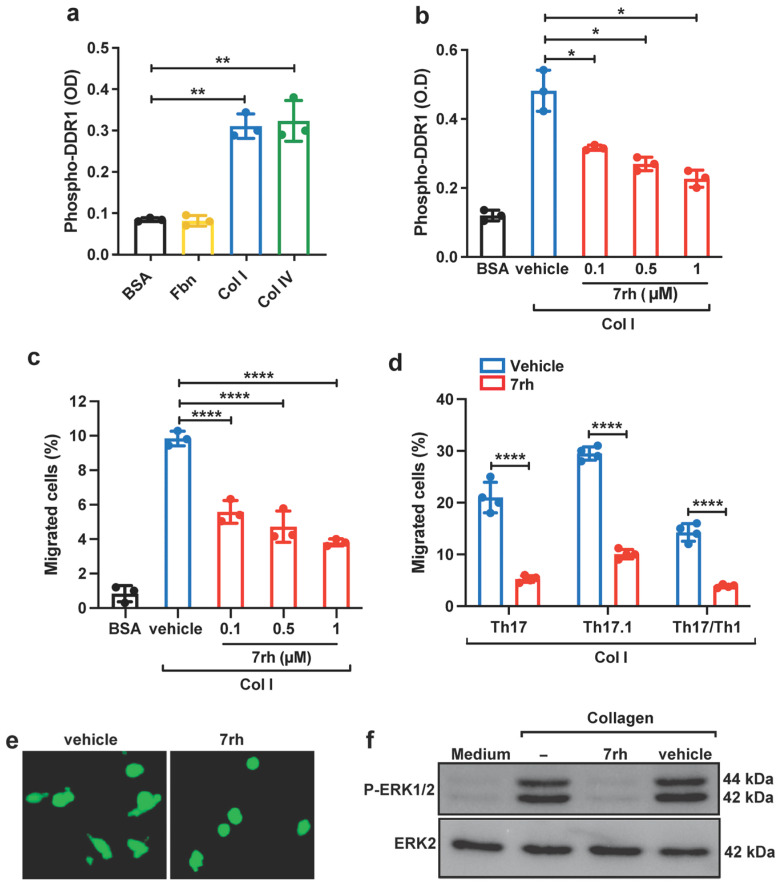
The DDR1 kinase activity promotes the migration of human CD161^+^CCR6^+^CD25^−^ effector/memory Th17 cells in 3D collagen. (**a**) Collagen stimulation induces DDR1 activation. The cells taken after 4 days of in vitro expansion were activated for 1 h with either 25 μg/mL BSA, fibronectin (Fbn), collagen I (Col I), or collagen IV (Col IV). Afterwards, they were lysed and DDR1 tyrosine phosphorylation was determined by ELISA. (**b**) The DDR1 kinase inhibitor 7rh reduces DDR1 phosphorylation. Cells were pre-treated for 1 h either with vehicle or increasing concentrations of 7rh. The cells were washed and activated or not with collagen I (Col I) for 1 h, after which they were lysed and DDR1 tyrosine phosphorylation was determined by ELISA. (**c**) The DDR1 inhibitor 7rh reduces the migration of human effector/memory CD161^+^CCR6^+^CD25^−^ Th17 cells in collagen gels (Col I). Cells were pre-treated for 1 h either with vehicle or increasing concentrations of 7rh, washed and seeded on collagen gel-coated inserts. After 24 h, cells that migrated to the outer wells were harvested and counted. (**d**) The DDR1 inhibitor 7rh reduces the migration of all three Th17 cell subsets. Human effector/memory CD161^+^CCR6^+^CD25^−^ Th17 cells were treated with either vehicle or 7rh and then subjected to collagen gel (Col I) migration assay. Migrated cells were recovered and counted and the proportion of Th17 subsets that migrated was determined by intracellular staining of IFN-γ and IL-17 and FACS analysis. (**e**) 7rh inhibits the migratory morphology of human effector/memory Th17 cells in collagen gels. Representative confocal photography images are from three independent experiments (400× magnification). (**f**) The DDR1 kinase is necessary for collagen-induced MAPK/ERK activation. Cells were pre-treated for 1 h either with vehicle or 1 μM of 7rh and then activated for 1 h with collagen. ERK phosphorylation was determined by immunoblot as described in the Supplementary Materials. The blot is representative of three different experiments. Results represent mean values ± SD of three to four different experiments performed in triplicates with Th17 cells isolated from three to four different blood donors. * *p* < 0.05; ** *p* < 0.01; **** *p* < 0.0001 calculated using one-way (**a**–**c**) or two-way (**d**) ANOVA with Bonferroni correction.

Next, we investigated whether the DDR1 tyrosine kinase is required for DDR1 function. To this end, we studied in vitro expanded CCR6^+^CD161^+^CD25^−^ Th17 cells since they contain solely effector/memory-DDR1 positive cells and are in large numbers. Activation of these cells with collagen types I and IV, two DDR1 ligands, led to increased tyrosine phosphorylation of DDR1 (Figure 2a). However, treatment of the cells with BSA or fibronectin, a major ECM component that does not bind DDR1, did not affect DDR1 phosphorylation. These results indicate that human effector/memory CCR6^+^CD161^+^CD25^−^ Th17 cells express a functional DDR1.

To assess the implication of the DDR1 kinase activity, we evaluated the effect of the selective DDR1 kinase inhibitor 7rh, which has been extensively used in DDR1 studies [36,37,38,39]. The 7rh dose-dependently reduced collagen-induced DDR1 tyrosine phosphorylation (Figure 2b) and the migration of effector/memory CCR6^+^CD161^+^CD25^−^ Th17 cells in collagen gels (Figure 2c). Th17 cell migration is inhibited by 65% when 7rh was used at 0.5–1 μM. In addition, the migration of all three Th17 cell populations, including Th17, Th17/Th1 and Th17.1, is inhibited by 7rh (Figure 2d). Although 7rh can inhibit other kinases especially Src kinases, we found that under our experimental conditions, 7rh had no effect on Lck activation in human Th17 cells suggesting the selectivity of 7rh in our model. Treatment with 7rh also inhibited the roundish/ellipsoid migratory shape of Th17 cells that characterizes the amoeboid movement of activated T cells in 3D collagen (Figure 2e). Like ex vivo Th17 cells, treatment of human polarized Th17 cells with 7rh reduced collagen-induced DDR1 phosphorylation and migration (Figure S3), and additional DDR1 kinase inhibitors imatinib and nilotinib also inhibited human Th17 cell migration in collagen (Figure S4). Finally, we tested if the DDR1 kinase is important for Th17 cell migration in the presence of chemokines. Th17 cells express the chemokine receptor CCR6 and respond to the chemokine CCL20. As we previously reported [23], CCL20 increases the number of Th17 cells migrating through collagen gels (Figure S5). Importantly, 7rh also inhibited Th17 cell migration in response to CCL20 (Figure S5), suggesting the importance of DDR1 kinase in Th17 cell migration in collagen. The 7rh had no effect on cell viability or proliferation for up to 48 h of treatment and also did not affect the production of IL-17 by Th17 cells.

To further examine the role of the DDR1 kinase in Th17 cell migration, we determined if it is required for the activation of MAPK/ERK, which we have previously reported to be essential for the migration of human polarized Th17 cells in collagen gels [23]. The results show that inhibition of the DDR1 kinase activity with 7rh blocked collagen-induced MAPK/ERK activation in human CD161^+^CCR6^+^CD25^−^ Th17 cells (Figure 2f), further supporting the role of the DDR1 kinase.

**Figure 3:**
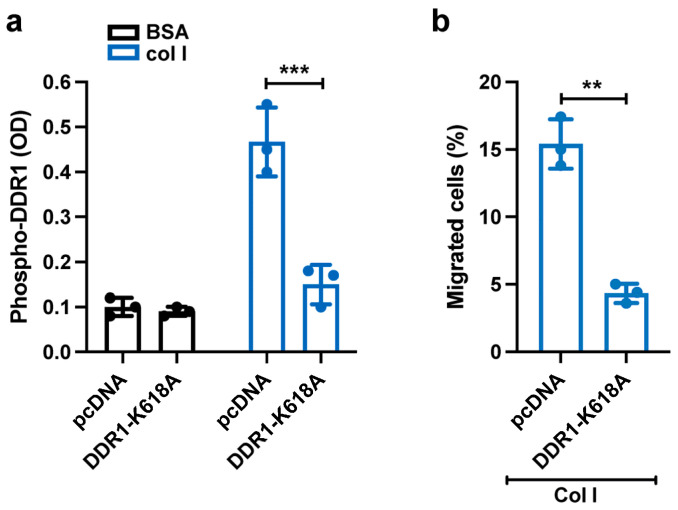
The DDR1 kinase-dead construct inhibits human Th17 cell migration in 3D collagen. Human effector/memory CD161^+^CCR6^+^CD25^−^ Th17 cells expanded in vitro for 4 days were transfected by nucleofector with either control (pcDNA) or DDR1-K618A kinase-dead plasmids. (**a**) Transfected cells were activated or not with collagen and DDR1 tyrosine phosphorylation was determined by ELISA. (**b**) Transfected cells were tested for their migration in collagen gels (Col I) and the number of migrated cells into the lower chambers was determined. Results represent mean values ± SD of three different experiments performed with Th17 cells isolated from three different blood donors. ** *p* < 0.01; *** *p* < 0.001 calculated using one-way ANOVA with Bonferroni correction (**a**) and Student’s *t*-test (**b**).

To confirm the effect of 7rh, we evaluated the effect of the DDR1 kinase dominant negative construct, DDR1-K618A. Transfection of human effector/memory CD161^+^CCR6^+^CD25^−^ Th17 cells with DDR1-K618A plasmid markedly reduced collagen-induced DDR1 tyrosine phosphorylation (Figure 3a) and Th17 cell migration in collagen gels (Figure 3b). As a control, the DDR1-K618A-transfected cells produce comparable amounts of IL-17 and IFN-γ as control transfected cells indicating that the DDR1 kinase-dead construct has not affected the function of Th17 cells (Figure S6). Together, these results demonstrate that DDR1 tyrosine kinase activity is required for human Th17 migration in 3D collagen.

### 2.3. DDR1 Promotes the Development of Collagen-Induced Arthritis (CIA)

**Figure 4:**
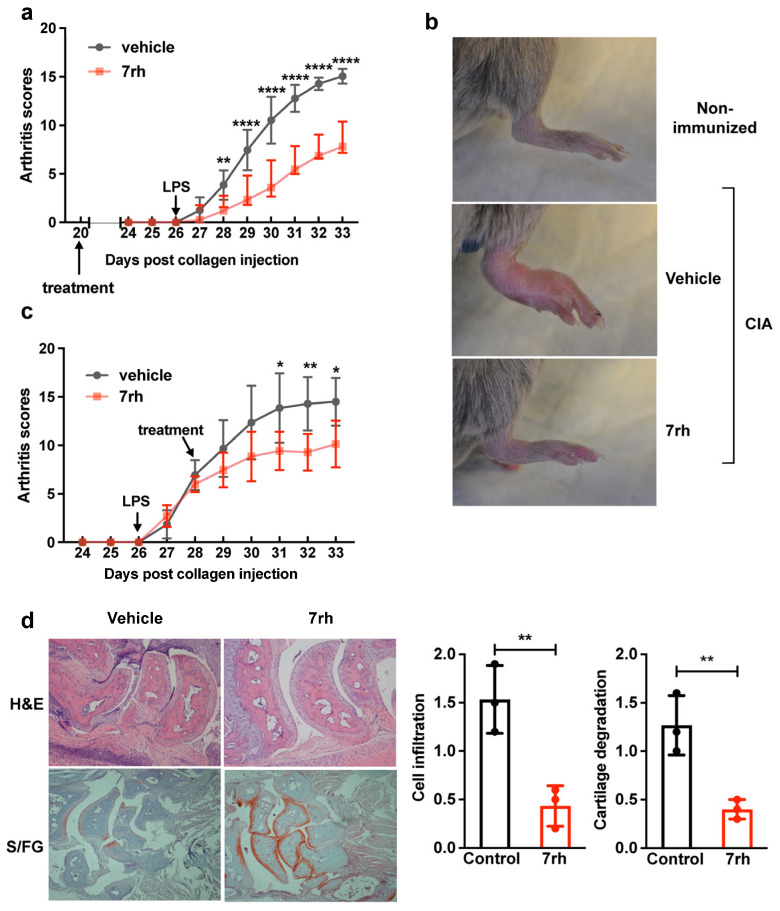
The DDR1 kinase inhibitor 7rh reduces the severity of CIA in mice. (**a**) Arthritis scores of vehicle- and 7rh-prophylactically treated CIA mice (*n* = 8) were assessed by two blinded observers. (**b**) Representative images of arthritic hind paws at day 33 of vehicle- and 7rh-treated CIA mice compared to non-immunized mice. (**c**) Arthritis scores of mice treated therapeutically with 7rh (*n* = 8). (**d**) Representative images of H&E- and Safranin O/Fast Green (S/FG)-stained sections of joints in the carpal regions from vehicle- and 7rh-treated arthritic mice (prophylactically) at 100× original magnification. Quantification of cell infiltration and cartilage degradation (histogram panels) (scale 0–2) in the carpal joints of arthritic mice (*n* = 3) was carried out as described in the Supplementary Materials. Three different fields/areas were scored and averaged for each mouse. Results represent mean values ± SEM. * *p* < 0.05; ** *p* < 0.01; **** *p* < 0.0001 calculated using two-way ANOVA with Bonferroni correction (**a**,**c**) and Student’s *t*-test (**d**).

Based on the results with human Th17 cells, we assessed whether the blockade of DDR1 with 7rh would regulate the development of CIA in mice, an arthritis model known to be driven by Th17 cells. Mice treated prophylactically with 7rh showed a marked decrease in arthritis clinical scores (Figure 4a,b), and a reduction in weight loss (Figure S7) compared to vehicle-treated mice. At day 33, the arthritic score was 50% lower in the 7rh-treated group compared to the vehicle-treated mice (Figure 4a). Importantly, mice treated therapeutically with 7rh showed, at days 31–33, a 30% reduction in arthritis scores compared to control mice (Figure 4c). Histological analysis of prophylactically treated mice revealed a significant decrease in synovial inflammation and cartilage degradation (Figure 4d). The 7rh reduced cell infiltration and cartilage degradation by 60% and 70% respectively (Figure 4d, right panels). Taken together, these results indicate that DDR1 inhibition reduced the severity of arthritis in the CIA mouse model.

### 2.4. DDR1 Inhibition Reduces T Cell Migration into the Arthritic Joints

**Figure 5:**
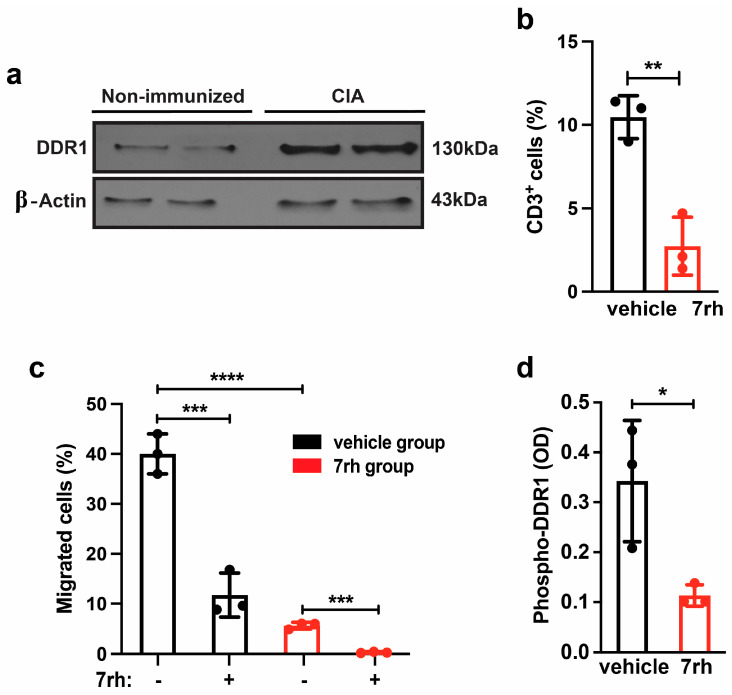
The DDR1 kinase inhibitor 7rh reduces T cell infiltration in arthritic mice. (**a**) DDR1 is highly expressed in T cells from arthritic mice compared to non-immunized mice. Total cell suspensions were prepared from lymph nodes and spleens of arthritic (CIA) and non-immunized mice. CD3^+^ T cells were FACS-sorted, and DDR1 expression was assessed by immunoblot analysis using anti-DDR1 antibody (D1G6). The blot was stripped and re-probed with an anti-β-actin antibody as a loading control. Results are representative of two different experiments. (**b**) The 7rh inhibitor reduces the number of T cells infiltrating the joints. Total cell suspensions from the joints of vehicle- and 7rh-treated CIA mice were stained with either control isotype or anti-CD3 antibodies and analyzed by FACS. (**c**) Treatment of arthritic mice with 7rh reduces the migratory capacity of arthritic T cells in collagen gels. CD3^+^ T cells were FACS-sorted from lymph nodes and spleens of vehicle- and 7rh-treated CIA mice and were pre-treated for 1 h with either vehicle or 7rh (1 µM), washed and seeded onto collagen gel-coated inserts. After 24 h, migrated cells were collected from the lower chamber and counted. (**d**) 7rh reduces DDR1 activation in arthritic joints. Lysates from arthritic joints were prepared as described in the Materials and Methods section and DDR1 phosphorylation was assessed by ELISA. Results represent mean values ± SEM (*n* = 3). * *p* < 0.05; ** *p* < 0.01; *** *p* < 0.001, **** *p* < 0.0001 calculated using Student’s *t*-test (**b**,**d**) and two-way ANOVA with Bonferroni correction (**c**).

To examine whether DDR1 promoted CIA by enhancing T cell migration into the joints, we first determined if DDR1 is associated with arthritic T cells, which we assessed by immunoblot, as an anti-mouse DDR1 antibody that works in FACS is not available. FACS-sorted T cells from arthritic mice express higher levels of DDR1 than those from non-immunized mice (Figure 5a). Accordingly, treatment with 7rh drastically reduced the number of T cells infiltrating the arthritic joints (Figure 5b and Figure S8).

To assess if the effect of 7rh on T cell infiltration was due to altered migration, FACS-sorted T cells from arthritic mice were tested for their ability to migrate in collagen gels. The results indicate that arthritic T cells from vehicle-treated mice have a far superior capacity to migrate in collagen gels than those isolated from 7rh-treated mice (Figure 5c). In addition, 7rh treatment further reduced the ability of T cells isolated from both vehicle- and 7rh-treated arthritic mice to migrate in collagen gels.

Finally, analysis of cell lysates from the arthritic joints revealed a 70% reduction in DDR1 phosphorylation in 7rh-treated samples compared to vehicle-treated samples (Figure 5d), suggesting that 7rh treatment acted through its target.

**Figure 6:**
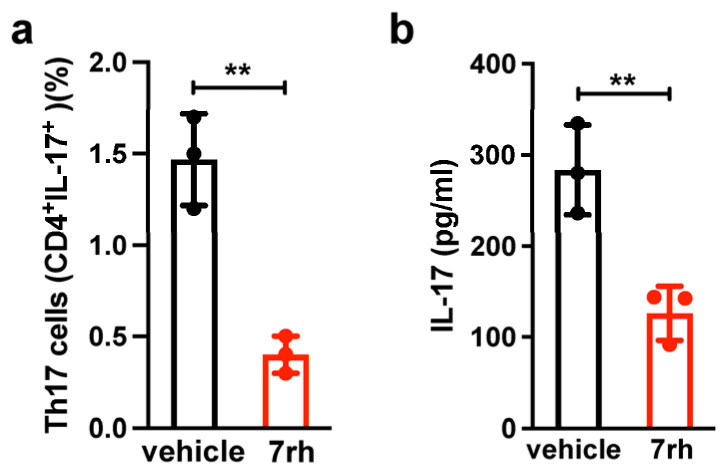
The DDR1 kinase inhibitor 7rh decreases Th17 cell activity in the joints of arthritic mice. Total cell suspensions prepared from the joints of vehicle- and 7rh-treated CIA mice were stimulated for 6 h in complete RPMI with PMA and ionomycin in the presence of brefeldin A. Then they were first stained with Alexa Fluor 647-conjugated anti-CD4 followed by intracellular staining with Alexa Fluor 488-conjugated anti-IL-17 antibodies. The cells were also stained with control isotype antibodies. The cells were washed and analyzed by flow cytometry. Th17 cells were identified as the CD4^+^/IL-17^+^ cell population. (**a**) Histogram represents the percentages of total Th17 cells (CD4^+^/IL-17^+^) in the joints of vehicle- and 7rh-treated arthritic mice. (**b**) Treatment with 7rh decreases IL-17 levels in arthritic joints. Lysates from the joints of vehicle- and 7rh-treated arthritic mice were prepared as described in the Materials and Methods section, and IL-17 levels were measured by ELISA. Results represent mean values ± SEM (*n* = 3). ** *p* < 0.01 calculated with Student’s *t*-test.

To determine if 7rh affected Th17 cell infiltration into the joints, we examined Th17 cell number in the joints of vehicle- and 7rh-treated arthritic mice. The results showed that 7rh reduced the number of Th17 cells infiltrating the joints (Figure 6a). The 7rh reduced the number of Th17 cells (CD4^+^IL-17^+^) by 65% (Figure 6a). Consistent with the reduction in the number of IL-17^+^ cells, 7rh also decreased the levels of IL-17 in the arthritic joints (Figure 6b). Taken together, these results indicate that DDR1 inhibition with 7rh protects the mice from arthritis at least by reducing Th17 cell infiltration into the joints.

### 2.5. 7rh Reduces Inflammatory Cytokines and Increases IL-10 Regulatory Cytokine

**Figure 7:**
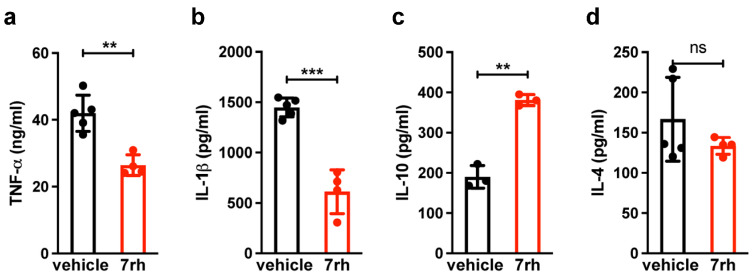
The DDR1 kinase inhibitor 7rh decreases TNF-α and IL-1β levels while increasing IL-10 levels in arthritic joints. Lysates from the joints of vehicle- and 7rh-treated arthritic mice were prepared and the concentration levels of TNF-α (**a**), IL-1β (**b**) IL-10 (**c**), and IL-4 (**d**) were measured by ELISA. Results represent mean values ± SEM (*n* = 5). ** *p* < 0.01, *** *p* < 0.001 calculated using Student’s *t*-test. ns: not significant.

In addition to IL-17 levels, treatment of arthritic mice with 7rh led to approximately 40% and 65% reduction in TNF-α (Figure 7a) and IL-1β levels (Figure 7b), respectively. In contrast, 7rh led to a two-fold increase in the levels of the immunoregulatory cytokine IL-10 in arthritic joints (Figure 7c), whereas the levels of IL-4 were unchanged (Figure 7d). These results indicate that DDR1 kinase inhibition promoted an anti-inflammatory response in the arthritic joints.

## 3. Discussion

The interaction of effector T cells with collagen is essential for their migration and persistence within tissues and is likely to play a crucial role in the development of anti-microbial immunity and inflammatory diseases. However, the mechanisms by which effector T cells migrate in tissue ECM are not fully elucidated. Here we found that the DDR1 tyrosine kinase activity is a critical pathway for the migration of human Th17 cells in collagen gels and into the joints during collagen-induced arthritis in mice. The present study revealed that DDR1 is a pathogenic pathway in the development of autoimmune arthritis.

We found that DDR1 expression is associated with human effector but not central/memory Th17 cells, suggesting an important role for DDR1 in the trafficking of effector T cells into inflammatory tissues in which they are likely to encounter collagen. However, central/memory T cells, which recirculate from the blood into lymph nodes, are unlikely to encounter collagen, as most of the collagen fibers in lymph nodes are surrounded by reticular fibroblasts and likely not accessible to T cells. Accordingly, human central/memory Th17 cells are poorly migratory in collagen gels compared to effector/memory Th17 cells.

Previous studies showed that DDR1 tyrosine kinase activity is not always required for its function [24,25,26]. Our findings indicate that the DDR1 tyrosine kinase activity is essential for Th17 cell migration both in vitro and in vivo. Our results suggest that it can do so likely through MAPK/ERK activation, which we have previously shown to be critical for amoeboid migration of human polarized Th17 cells in 3D collagen [23]. In agreement, the MAPK/ERK pathway has recently been involved in the amoeboid movement of metastatic cancer cells [40]. Thus, the DDR1 kinase activity promotes Th17 cell amoeboid migration at least by enhancing MAPK/ERK activation.

It has been reported that the DDR1 tyrosine kinase activity is required to induce the motility of breast cancer cells on collagen by enhancing cell contraction through activation of the non-muscle myosin II [41]. This is in accordance with our findings since Th17 cells migrate in 3D collagen via an amoeboid movement [23], which is also dependent on cell contraction and myosin II activation. Although myosin II activation has not been examined, our study strongly supports the implication of DDR1 kinase in Th17 cell contraction during their migration in 3D collagen.

The use of the selective DDR1 kinase inhibitor 7rh demonstrated that DDR1 is critical for the development of arthritis in the CIA mouse model. Our results showed that DDR1 is expressed on autoreactive T cells, and its inhibition reduced the migration of total T cells and of Th17 cells into the joints, which is correlated with the reduced ability of 7rh-treated arthritic T cells to migrate in collagen gels. These results indicate that DDR1 kinase contributes to arthritis, likely by enhancing the capacity of effector T cells to migrate through tissue collagen and to localize into the inflammatory joints.

DDR1 is a major mediator of inflammation and fibrosis in animal models of renal and pulmonary diseases [42,43,44]. The role of DDR1 in these models is associated with tissue infiltration of both T cells and macrophages, but only macrophages express high levels of DDR1 [43,44]. This contrasts with our study in which arthritic T cells do express DDR1. This might be because the models of renal and pulmonary diseases are not autoimmune diseases in nature and do not rely on antigen-induced T cell activation and persistence of autoreactive T cells, as is the case with the CIA model. Along these lines, DDR1 is induced during antigenic activation of human T cells [20,21,22]. It is also possible that DDR1 is associated with specific T cell subsets. The CIA model is mainly driven by Th17 cells whereas the renal and pulmonary fibrosis models cited above are dependent on Th2 cells [45,46,47]. In this context, our results showed that the treatment with 7rh did not alter the levels of the Th2 cytokine IL-4 in the arthritic joints, suggesting that Th2 cells may be independent of DDR1. Importantly, human polarized Th2 cells express weak levels of DDR1 compared to human Th17 cells (Figure S9), which is in line with findings from renal and pulmonary fibrosis inflammatory models.

DDR1 has been reported to be expressed on macrophages in animal models of fibrosis [43,44] and cardiometabolic disease [16] and in chondrocytes as well [39]. These cells also play an important role in arthritis and in the CIA model. Therefore, it is not excluded that the inhibitory effect of 7rh seen in the CIA model could also be exerted on these cells, thus contributing to reduction in arthritis severity. However, given that Th17 cells are critical in the CIA model and are known to upregulate the function of macrophages, chondrocytes and other inflammatory cells [48], it is likely that they represent a major target for 7rh and the reduction in arthritis severity. This is supported by the results showing that T cells isolated from 7rh-treated CIA mice are less migratory in collagen gels than those from vehicle-treated CIA mice (Figure 5c). Thus, our study indicates that Th17 cells are dependent on DDR1 for their pathogenic function in autoimmune arthritis.

DDR1 has recently been associated with the development of osteoarthritis [39], which is not considered an immune-mediated disease and therefore DDR1 may be a general pathogenic pathway in rheumatic diseases. Our study showed that treatment of mice with 7rh in a therapeutic setting also attenuated CIA, suggesting that DDR1 can represent a therapeutic target and the DDR1 inhibitors are potential novel drugs for the treatment of RA. However, we acknowledge that peripheral blood Th17 cells may differ phenotypically and functionally from autoreactive RA T cells. Likewise, the CIA model does not fully reproduce the heterogeneity and chronicity of human RA. Therefore, while the concordant findings in human cells and the murine model strengthen the translational relevance of our observations, additional studies with T cell samples from RA patients and other mouse models will be required to fully establish whether DDR1 is a therapeutic target in RA. In addition, future studies are needed to establish the efficacy of potential DDR1 inhibitors in comparison with current therapies in RA.

## 4. Materials and Methods

### 4.1. Reagents and Antibodies

The reagents and antibodies used in this study are listed in the Supplementary Materials (Supplemental Table S1).

### 4.2. Human Polarized Th17 Cells, Isolation of CD4^+^CD45RO^+^CD161^+^CCR6^+^CD25^−^ Th17 Cells and DDR1 Expression

To obtain polarized Th17 cells, naïve CD4^+^ T cells were purified from the peripheral blood of healthy donors using an EasySep isolation kit (STEMCELL Technologies, Vancouver, Vancouver, BC, Canada) and activated for 4 days with anti-CD3/CD28 beads in the presence of polarizing cytokines (IL-6, TGF-β, IL-1β, and IL-23) as we previously described [49,50]. The CD4^+^CD45RO^+^CD161^+^CCR6^+^CD25^−^ T cells comprising Th17, Th17/Th1 and Th17.1 subsets, and referred to hereafter as CD161^+^CCR6^+^CD25^−^ Th17 cells, were isolated from the peripheral blood of healthy adult volunteers through cell sorting and flow cytometry (Supplementary Materials). Both male and female donors were studied and no differences in DDR1 expression, Th17 migratory capacities and 7rh responses were noted between the two sexes.

DDR1 expression was assessed by flow cytometry. CD161^+^CCR6^+^CD25^−^ Th17 cells were first labeled with 10 µg/mL of unconjugated mouse anti-DDR1 (clone: 5D5) or with appropriate isotype control antibodies. The cells were washed and stained with Alexa Fluor 647-conjugated goat anti-mouse secondary antibody (clone: ab150115). Labeled cells were washed and analyzed by flow cytometry using the BD FACSCanto II cytometer.

### 4.3. DDR1 Activation in Human CD161^+^CCR6^+^CD25^−^ Th17 Cells

DDR1 tyrosine kinase activation was assessed by ELISA using the PathScan Phospho-DDR1 (pan-Tyr) Sandwich ELISA kit (Cell Signaling Technology, Beverly, MA, USA) as previously described [23,51].

### 4.4. Collagen Gel and Th17 Cell Migration

Th17 cell migration in 3D collagen was performed using transwell inserts of polycarbonate membrane (3 μm, BD Biosciences, Milpitas, CA, USA) coated with collagen gels and mounted in 24-well plates. Collagen gel was prepared by diluting type I rat tail collagen (Corning ref:354236, Bradford, MA, USA) to a final concentration of 1.6 mg/mL in PBS and by adjusting the pH to 7.3 with NaOH. The collagen solution was overlaid on the inserts, which were incubated for 1 h at 37 °C to allow collagen polymerization. Cell suspensions (5 × 10^5^ cells in 350 μL of RPMI medium) were then added on top of the collagen gels. After 24 h, cells that had passed through the transwells to the other side of the filters and in the outer wells were recovered and counted microscopically by two blinded observers. Th17 cell shape in collagen gels was visualized by confocal microscopy (Supplementary Materials).

### 4.5. Plasmids and Th17 Cell Transfection

The plasmid encoding the kinase-dead form of DDR1 in which a lysine in the catalytic domain was mutated to alanine (K618A) was previously described [52] and was a gift of the late Dr Wolfgang Vogel (University of Toronto). DDR1-K618A or empty control (pcDNA) plasmids were transfected into in vitro expanded human CD161^+^CCR6^+^CD25^−^ effector/memory Th17 cells by the nucleofector method as we previously described [23].

### 4.6. Induction, Treatment, and Assessment of Collagen-Induced Arthritis (CIA)

CIA in DBA1/J male mice of 6–8 weeks old was performed as we previously described [50,53]. Mice were immunized s.c. (day 0) at the base of the tail with chicken collagen II emulsified in CFA (100 μg/mouse). On day 26 after immunization and to synchronize the onset of disease, mice were injected i.p with LPS (25 µg/mouse). Six days before LPS injection (prophylactic) or two days after the onset of arthritic symptoms (therapeutic), mice were treated daily with DDR1 kinase inhibitor (7rh) (7% DMSO, 15% Ethanol, 15% Kolliphor EL and 63% of H_2_O) administered by oral gavage at 35 mg/kg or with the vehicle as a control. The treatment lasted until the end of the experimental protocol. Arthritis scores and histological analysis were as we previously described [50,53] and in the Supplementary Materials.

### 4.7. Detection of DDR1 in Arthritic T Cells

Cellular suspensions from the lymph nodes and spleens of non-immunized and arthritic mice were prepared and stained with anti-CD3 antibody. After staining, the cells were washed and CD3^+^ T cells were FACS-sorted using an Aria fusion cytometer (BD Biosciences) and recovered in 5ml of complete RPMI. T cell purity and viability after cell sorting were over 97%. The cells were lysed, and DDR1 expression was determined by immunoblot using the DDR1 antibody (clone: D1G6). The blot was stripped and re-probed with an anti-mouse β-actin antibody. Protein bands were detected using the enhanced chemiluminescence substrate kit (PerkinElmer, Shelton, CT, USA).

### 4.8. Flow Cytometry of Mouse Th17 Cells

Cell suspensions were prepared from the hind paws of arthritic mice. Skin-free arthritic paws were rapidly incubated in PBS containing 3 mg/mL collagenase D (Roche Diagnostics GmbH, Mannheim, Germany) for 3 h at 37 °C with gentle agitation. The resulting material was then passed through 40 μm cell strainers to remove undigested tissues. T cells and Th17 cells were quantified with CD3 staining, and double staining with CD4 and IL-17, respectively, and analyzed by flow cytometry (Supplementary Materials).

### 4.9. DDR1 Activation and Cytokine Quantification in Arthritic Joints

Hind paws were removed and homogenized on ice in a tissue grinder (Polytron, Kinematica AG, Malters, Switzerland) in PBS containing the protease inhibitor mixture and phosphatase inhibitors (Roche, Basel, Switzerland). Samples were then sonicated (Branson Sonifier 450, Brookfield, CT, USA) on ice for 2 s at power level 1, and the lysates were cleaned by two successive centrifugations at 4000× *g* (5 min at 4 °C). Supernatants were recovered and protein levels were assessed using the Pierce^TM^ BCA Protein Assay Kit (Thermoscientific, Rockford, IL, USA). DDR1 tyrosine kinase activity was measured with the Sandwich PathScan Phospho-DDR1 (pan-Tyr) ELISA kit (Cell Signaling Technology, Beverly, MA, USA) [23,51]. Lysates were also subjected to ELISA for the detection of IL-17, IL-1β, IL-4, IL-10 and TNF-α cytokines using specific ELISA kits (R&D Systems, Minneapolis, MN, USA).

### 4.10. Statistical Analysis

Statistical analysis was performed using GraphPad Prism software version 10.4.2 Mac (Boston, MA, USA, www.graphpad.com). The data were analyzed with Student’s *t*-test for simple comparisons and with one-way or two-way ANOVA followed by Bonferroni correction for multiple comparisons. Differences between samples were considered statistically significant when the *p*-value was less than 0.05.

## Data Availability

The original contributions presented in this study are included in the article/Supplementary Materials. Further inquiries can be directed to the corresponding author.

## Supplementary Materials

The following supporting information can be downloaded at https://www.mdpi.com/article/10.3390/ijms27136043/s1.

## Abbreviations

The following abbreviations are used in this manuscript:

DDR	Discoïdin domain receptor
CIA	Collagen-induced arthritis
ECM	Extracellular matrix
RA	Rheumatoid arthritis
MAPK	Mitogen-activated protein kinase
ERK	Extracellular signal-regulated kinase
Th17	T helper 17 cells
Col	Collagen
