# Supplementary material for: The DDR1 Tyrosine Kinase Promotes Th17 Cell Migration in Three-Dimensional Collagen and into the Joints During Inflammatory Arthritis
Source: Int J Mol Sci. 2026 Jul 6;27(13):6043. doi: 10.3390/ijms27136043 (PMC13360736; doi:10.3390/ijms27136043)
Supplement: Supplementary file 1 [file ijms-27-06043-s001.zip › ijms-4362459-supplementary.pdf]

Supplementary Material

The DDR1 Tyrosine Kinase Promotes Th17 Cell Migration in Three-Dimensional Collagen and into the Joints During Inflammatory Arthritis

Chakib Hamoudi[1,2], Mehdi Toghi[1,2], Anahita Lashgari[1,2], Fawzi Aoudjit[1,2,3,*]

1 Division of Infectious and Immune Diseases, CHU de Quebec-Université Laval Research Center, CHUL, Quebec, QC G1V 4G2, Canada
2 ARThrite Research Center, Laval University, Quebec, QC G1V4G2, Canada
3 Department of Microbiology-Infectiology and Immunology, Faculty of Medicine, Laval University, Quebec, QC G1V 0A6, Canada
* Correspondence: fawzi.aoudjit@crchudequebec.ulaval.ca; Tel.: +1-418-525-4444 (ext. 46071)

Supplemental Table S1: List of antibodies and reagents used in this study.

Antibodies or reagents	Clone	Company
PE-Cy 5 mouse anti-human CD161	DX12	BD Bioscience (San Diego, CA, USA)
FITC mouse anti-human CD25	M-A251	BD Bioscience (San Diego, CA, USA)
Alexa Fluor 647 mouse anti-human CD196 (CCR6)	11A9	BD Bioscience (San Diego, CA, USA)
PE Rat anti-human CCR7 (CD197)	3D12	BD Bioscience (San Diego, CA, USA)
Alexa Fluor 647 mouse anti-human IL-17	N49-653	BD Bioscience (San Diego, CA, USA)
PE mouse anti-human IFN-γ	B27	BD Bioscience (San Diego, CA, USA)
Alexa Fluor 647 Rat anti-mouse CD4	RM4-5	BD Bioscience (San Diego, CA, USA)
PE Hamster anti-mouse CD3e	145-2C11	BD Bioscience (San Diego, CA, USA)
Alexa Fluor 488 Rat anti-mouse IL-17A	TC11-18H10	BD Bioscience (San Diego, CA, USA)
FC Block	2. 4G2	BD Bioscience (San Diego, CA, USA)
PE-Cy 5 mouse IgG1 κ Isotype Control	MOPC-21	BD Bioscience (San Diego, CA, USA)
FITC mouse IgG1 κ Isotype Control	MOPC-21	BD Bioscience (San Diego, CA, USA)
Alexa Fluor 647 mouse IgG1 κ Isotype Control	MOPC-21	BD Bioscience (San Diego, CA, USA)
PE mouse IgG1 κ Isotype Control	MOPC-21	BD Bioscience (San Diego, CA, USA)
Alexa Fluor 488 mouse IgG1 κ Isotype Control	MOPC-21	BD Bioscience (San Diego, CA, USA)
Alexa Fluor 488 donkey anti-rabbit IgG (H+L)	A-21206	Thermo fischer sc (Waltham, MA, USA)
Anti-ERK2	C-14	Santa Cruz Biotehcnologies (Santa Cruz, CA)
Anti-phospho-ERK1/2	E-4	Santa Cruz Biotehcnologies (Santa Cruz, CA)
Anti-β-actin	C-2	Santa Cruz Biotehcnologies (Santa Cruz, CA)
Anti-CD167a/DDR1 mouse monoclonal	5D5	Millipore-Sigma (St. Louis, MO, USA)
IgG 1 negative control mouse monoclonal	1.E.2.2	Millipore-Sigma (St. Louis, MO, USA)
Alexa fluor 647 Goat anti-mouse secondary antibody	ab150115	Abcam (Cambridge, UK)
Anti-DDR1 XP® Rabbit mAb	D1G6	Cell Signaling (Berverly, MA, USA)
Rabbit IgG XP® Isotype Control mAb	DA1E	Cell Signaling (Berverly, MA, USA)
Rat-tail type I collagen, collagen IV and collagenase		Corning (Bedford, MA, USA)
Fibronectin		Sigma-Millipore (St. Louis, MO, USA)
DDR1 kinase inhibitor (7rh)		Sigma-Millipore (St. Louis, MO, USA)

Supplemental methods

Isolation of $CD161^+CCR6^+CD25^-$ Th17 cells

Memory $CD4^+$ T cells ($CD45RO^+$) were first purified from peripheral blood of healthy donors by negative selection using an appropriate isolation kit from STEMCELL Technologies (Vancouver, BC) and then stained with antibodies targeting CD161, CCR6 and CD25 either separately or in combination. The $CD161^+CCR6^+CD25^-$ Th17 cell population was FACS-sorted using an Aria fusion cytometer (BD Biosciences). In certain experiments, the CCR7 antibody was added to FACS-sort the effector/memory and central/memory $CD161^+CCR6^+CD25^-$ Th17 cells.

The $CD161^+CCR6^+CD25^-$ Th17 cells (more than 97% purity) were expanded with anti-CD3/CD28 beads (two beads/cell) and IL-2 (30 units/ml) for 4 days. Cell viability was superior to 97% both after cell sorting and in vitro expansion.

$CD161^+CCR6^+CD25^-$ Th17 cells ($5x10^5$) were activated with PMA+ionomycin in the presence of BD Golgi Plug (5 µg/ml) containing brefeldin A. Cells were washed, fixed and permeabilized with the CytoFix/CytoPerm kit (BD Biosciences) at 4°C for 20 min, followed by incubation for 40 min on ice with anti-IL-17 and anti-IFN-γ antibodies or with control isotype antibodies. After three washes, IL-17- and IFN-γ-positive cells were evaluated by flow cytometry using the BD FACSCanto II cytometer.

Confocal Microscopy

$CD161^+CCR6^+CD25^-$ Th17 cells were stained with 5 nM of calcein-AM in the dark at 37°C, embedded into collagen gels and observed using a spinning disk confocal microscope (Wave FX-Borealis-Leica DMI 6000B, Quorum Technologies) and a 10X objective (HC PL Apo NA 0.4) as we previously described [23].

ERK phosphorylation

The cells were activated or not for 1 h with collagen in the presence or absence of 7rh. ERK phosphorylation was determined by immunoblot analysis using the anti-phospho-ERK1/2 (clone: E-4) antibody as we previously described [23]. Blots were stripped and re-probed with ERK2 (clone: C-14) antibody to ensure equal loading. Protein bands were detected using the enhanced chemiluminescence substrate kit (PerkinElmer).

Assessment of collagen-induced arthritis

After collagen immunization and LPS injection, mice were closely monitored and scored daily for clinical symptoms of arthritis in a blinded fashion by two observers until the animals were sacrificed on days 33-34 (7-8 days after LPS injection). A scoring scale of 0 to 4 per paw (for a maximum score of 16 per mouse) was used as we previously described [50, 53]. Forepaws were recovered from sacrificed mice and fixed in 4% paraformaldehyde, decalcified in TBD-2 Thermo-Shandon solution (Thermo Fisher Scientific) for 10 days, and then embedded in paraffin. Sections (5 μm thick) were prepared and stained with hematoxylin&eosin (H&E) (Thermo Fisher Scientific) to assess synovial inflammation and cellular infiltration, and stained with Safranin O/Fast Green (S/FG; VWR International) to assess cartilage degradation by evaluating the presence and intensity of red staining as we previously described [50, 53]. Quantification was performed by two blinded observers on a scale of 0 to 2 (0: normal, 1: mild, 2: severe) from images of three different fields/areas representing joints in the carpal and proximal interphalangeal regions.

Staining and flow cytometry analysis of mouse T cells

Cellular suspensions prepared from the hind paws of arthritic mice were stained for 40 min at 4°C

with either anti-mouse CD3 or control isotype antibodies. The cells were washed and analyzed by flow cytometry using BD FACSCanto II cytometer.

For Th17 cells, cellular suspensions prepared from arthritic paws were stimulated with PMA+ionomycin in the presence of brefeldin-A. Cells were stained with an anti-CD4 antibody (clone RM4-5), washed, fixed, and permeabilized with the CytoFix/CytoPerm kit. Cells were then stained with anti-IL-17 antibody (clone: TC11- 18H10) and analyzed by flow cytometry. Cells were also stained with isotype antibodies and used as controls in flow cytometry analysis. Th17 cells were identified as $CD4^+$/$IL-17^+$ cells.

In all cases, total cells were stained with Live/Dead™ fixable violet dead cell stain (Thermo Fisher Scientific, Waltham, MA), and Fc receptors on cells were blocked by incubation with Fc Block (BD Biosciences). Total cells were gated using forward scatter (FSC) area versus side scatter area (SSC), then gated on single cells to exclude doublets using FSC area versus FSC height, followed by gating on live cells.

Supplemental figures

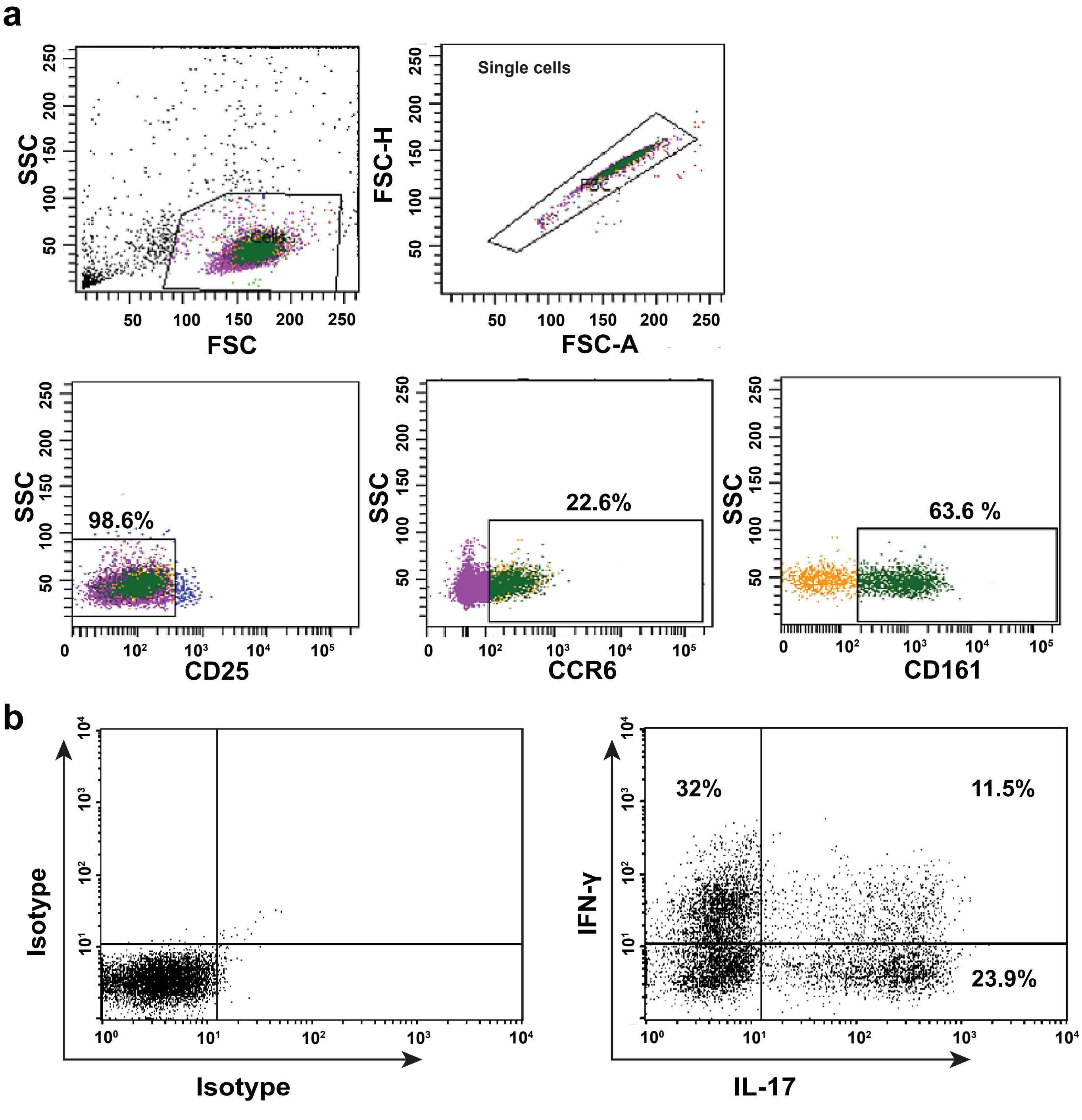

Figure S1. (a) Flow cytometry gating strategy used for the sorting of CD161$^+$CCR6$^+$CD25$^-$ Th17 cells from isolated peripheral blood CD4$^+$ memory T cells. Purity and viability are over 97%. **(b)** The cells produce IL-17 and IFN-γ after reactivation with PMA/ionomycin corresponding to Th17, Th17/ Th1 and Th17.1 cells. The results are representative of five experiments performed with different blood donors.

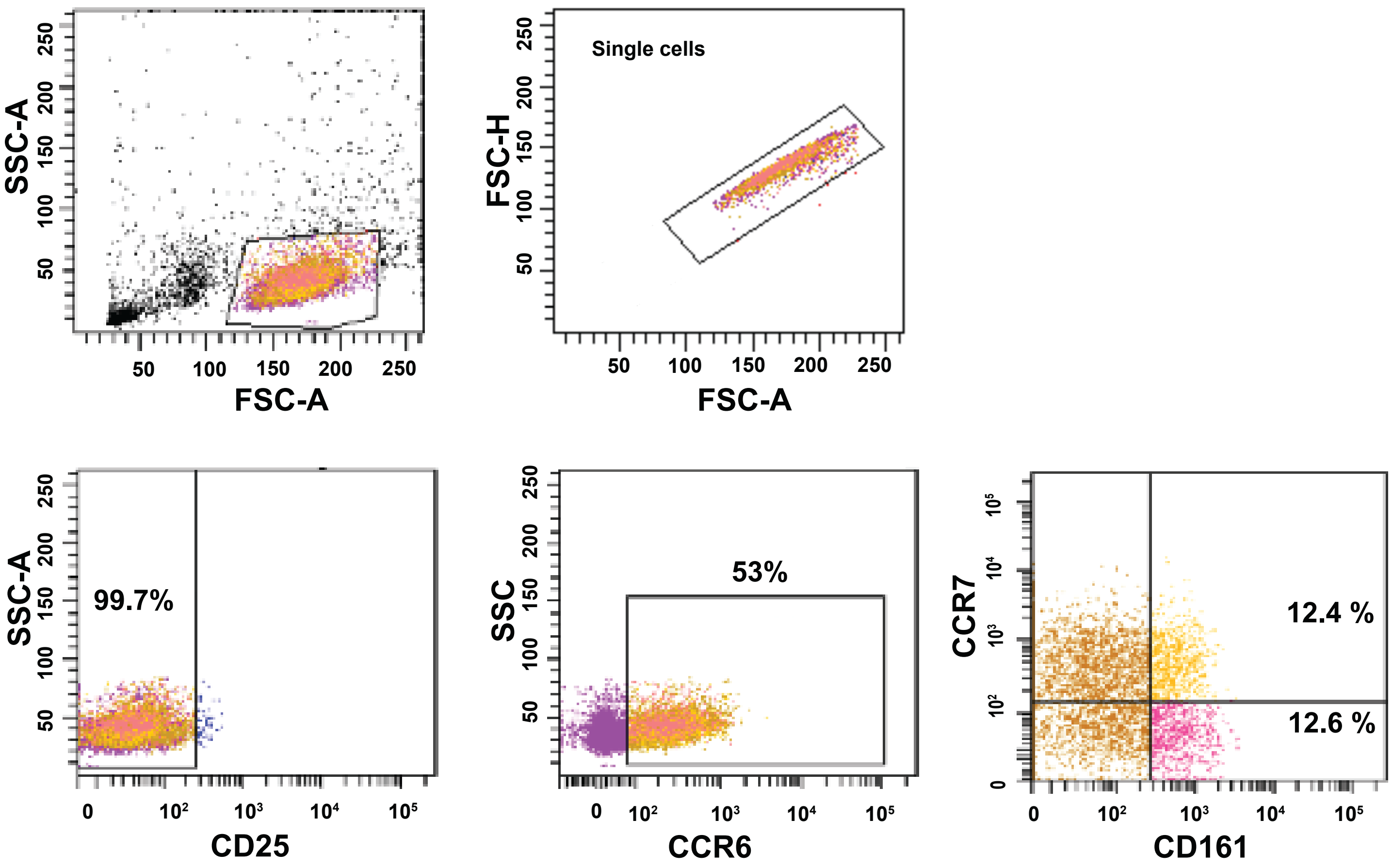

Figure S2. Flow cytometry gating strategy used for the sorting of effector/memory Th17 cells ($CD161^+$ $CCR6^+CD25^-CCR7^-$) and central/memory ($CD161^+CCR6^+CD25^-CCR7^+$) Th17 cells from isolated peripheral blood $CD4^+$ memory T cells. Purity and viability are over 97%.

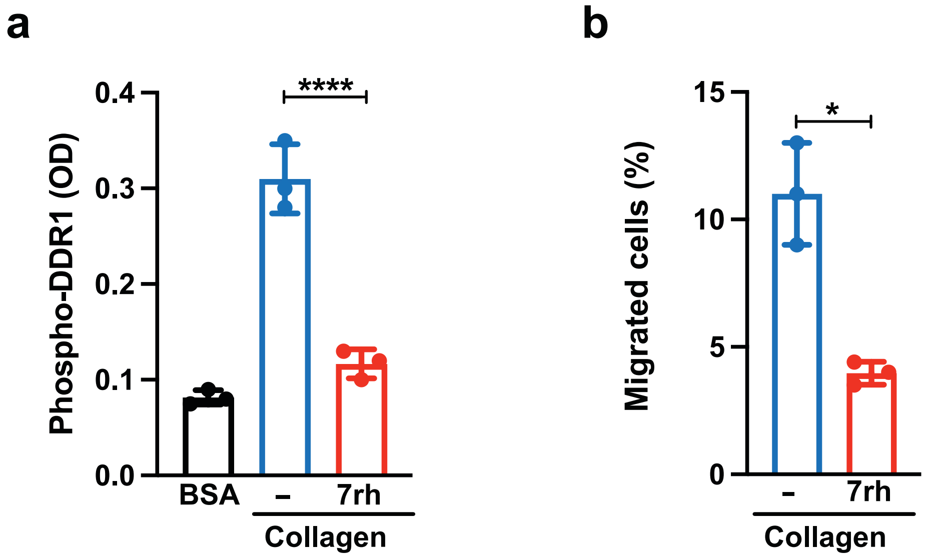

Figure S3. The DDR1 kinase inhibitor 7rh inhibits DDR1 activation **(a)** and migration **(b)** of human polarized Th17 cells in collagen gels. Results represent mean values ± SD of three different experiments performed in triplicates with T cells isolated from three different blood donors. *$p < 0.05$, ****$p < 0.0001$ calculated using one-way ANOVA with Bonferroni correction (panel a) and Student's t-test (panel b).

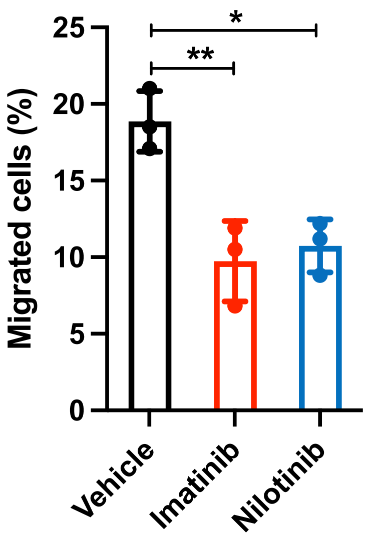

Figure S4. Imatinib and Nilotinib (1 µM) inhibit the migration of effector/memory CD161$^+$CCR6$^+$CD25$^-$ Th17 cells in collagen gels. The cells were tested after 4 days of in vitro expansion of FACS-sorted CD161$^+$CCR6$^+$ CD25$^-$ Th17 cells, as described in the Material and methods section. The results represent mean values ± SD of three different experiments performed in triplicates with T cells isolated from three different blood donors. *$p < 0.05$, **$p < 0.01$ calculated using one-way ANOVA with Bonferroni correction.

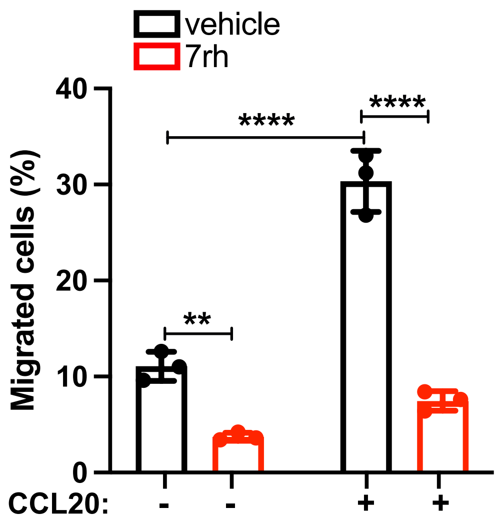

Figure S5. The DDR1 kinase inhibitor 7rh inhibits the migration of effector/memory CD161$^+$ CCR6$^+$CD25$^-$ Th17 cells in response to CCL20. Human effector/memory CD161$^+$CCR6$^+$CD25$^-$ Th17 cells expanded in vitro for 4 days were treated with vehicle or 7rh and then added on top of collagen gel-coated inserts. After 24 h, cells that had passed into the outer wells, which contained medium with or without 1 µg/ml of CCL20 were counted microscopically. Results represent mean values ± SD of three different experiments performed in triplicates with Th17 cells isolated from three different blood donors. **p < 0.01, ****p < 0.0001 calculated using one-way ANOVA with Bonferroni correction.

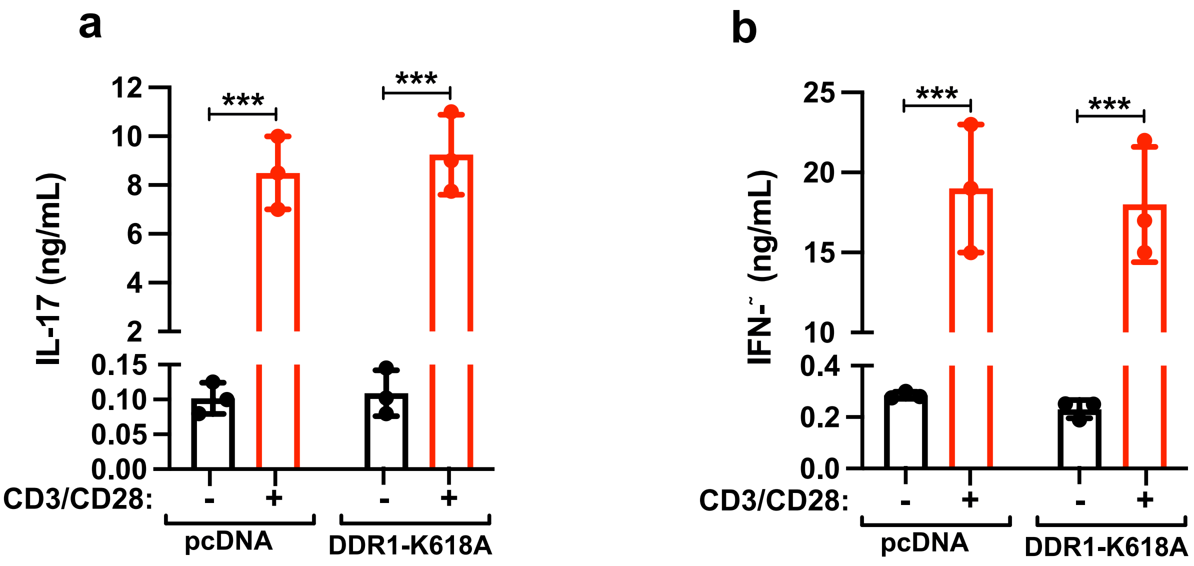

Figure S6. The DDR1-kinase dead construct does not affect human Th17 cell function. Human effector/memory CD161$^+$CCR6$^+$CD25$^-$ Th17 cells expanded in vitro for 4 days were transfected by nucleofector with either control (pcDNA) or DDR1-K618A kinase-dead plasmids. After transfection, the cells were activated with CD3/CD28 beads and the production of IL-17 (panel a) and IFN-γ (panel b) was determined by ELISA. Results represent mean values ± SD of three different experiments performed in triplicates with T cells isolated from three different blood donors. ***$p < 0.001$ calculated using one-way ANOVA with Bonferroni correction.

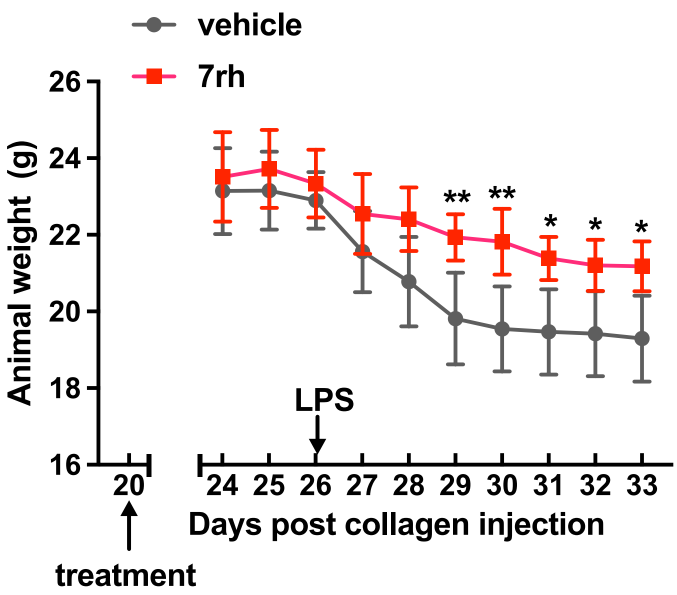

Figure S7. The DDR1 Kinase inhibitor 7rh mitigates CIA-induced weight loss in mice. Data are presented as mean values ± SEM (n= 8). *p < 0.05, **p < 0.01 calculated using two-way ANOVA with Bonferroni correction.

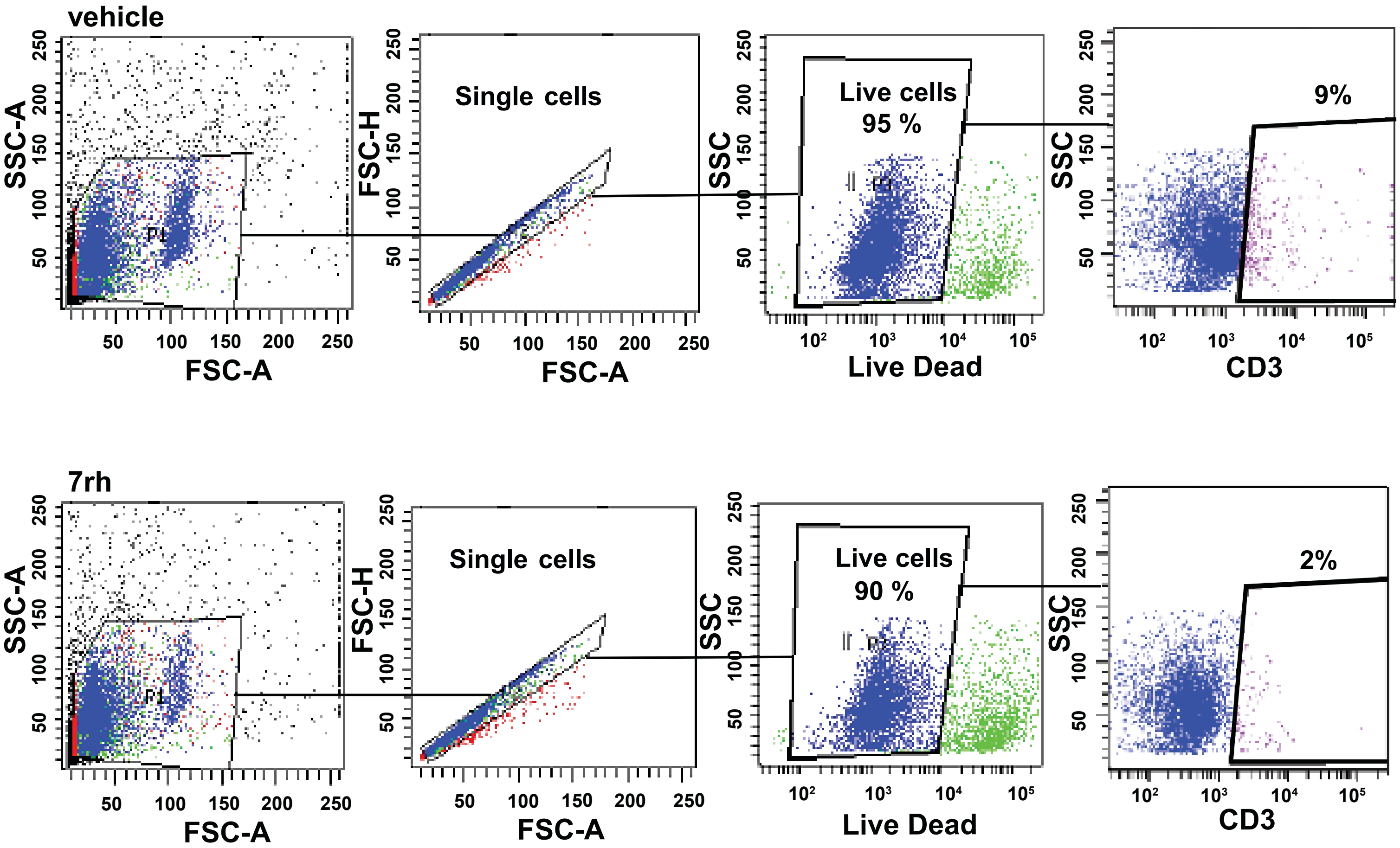

Figure S8. Flow cytometry gating strategy used for identifying $CD3^{+}$ cells in cellular suspensions prepared from hind paws of vehicle- and 7rh-treated arthritic mice.

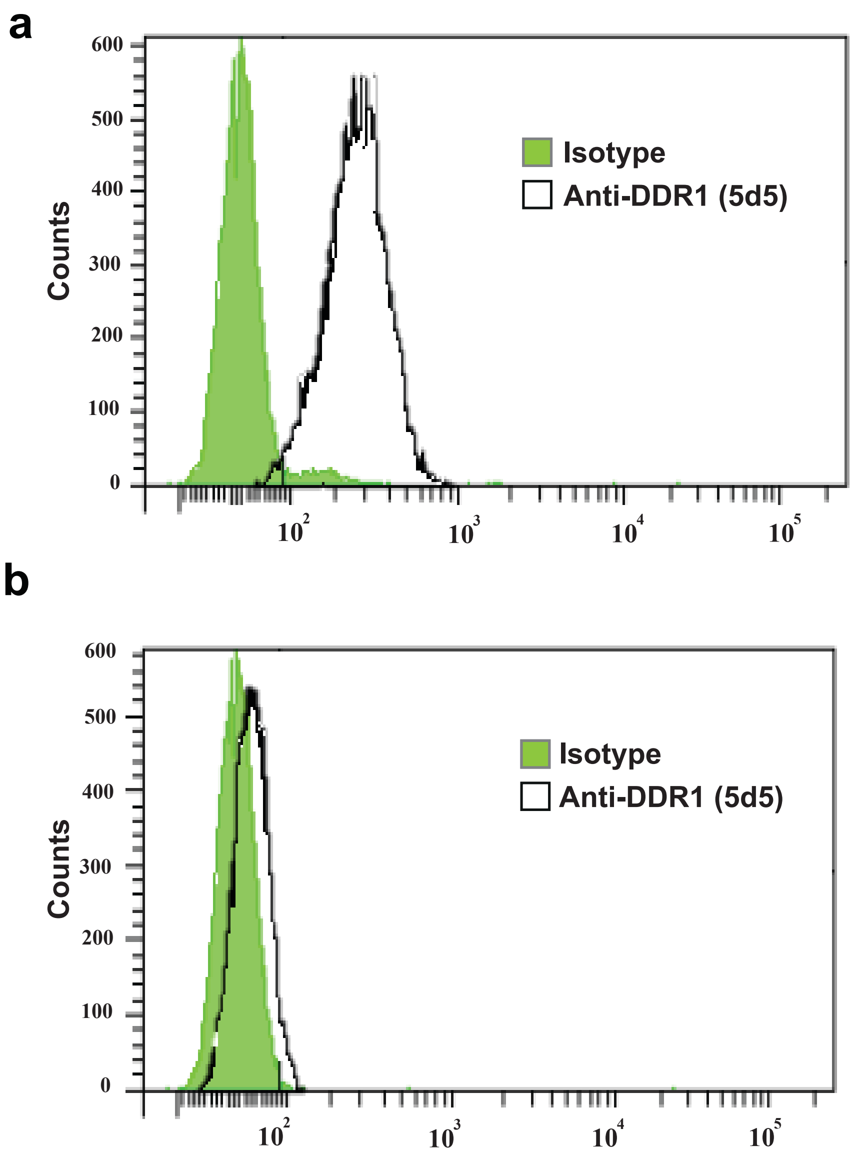

Figure S9. Polarized Th2 cells express weak levels of DDR1. (a) Human polarized Th2 cells express weak levels of DDR1. (a) Human polarized Th17 cells were generated as described in the Material and Methods section. (b) Polarized Th2 cells were generated after activation of naïve CD4+ T cells in X-vivo medium containing anti-CD3/CD28 beads, IL-2 (20 ng/ ml), IL-4 (20 ng/ml) and anti-IFN-γ antibody (2 µg/ml). After four days, the cells were labelled for DDR1 as described in the Materials and methods section. Labeled cells were analyzed by flow cytometry using the BD FACS Canto II cytometer.
